# Supplementary material for: The High-Temperature Soft Ferromagnetic Molecular Materials Based on [W(CN)6(bpy)]2−/− System
Source: Molecules. 2022 Jul 15;27(14):4525. doi: 10.3390/molecules27144525 (PMC9315671; doi:10.3390/molecules27144525)
Supplement: Supplementary file 1 [file molecules-27-04525-s001.zip › Supporting Information.pdf]

Supporting Information

The high-temperature soft ferromagnetic molecular materials based on $[W(CN)_6(bpy)]^{2-/-}$ system

Janusz Szklarzewicz[1*], Maciej Hodorowicz[1], Anna Jurowska[1] and Stanisław Baran[2]

[1] Faculty of Chemistry, Jagiellonian University, Kraków, Poland.

[2] M. Smoluchowski Institute of Physics, Faculty of Physics, Astronomy and Applied Computer Science, Jagi-ellonian University, Kraków, Poland.

* e-mail: janusz.szklarzewicz@uj.edu.pl, szklarze@chemia.uj.edu.pl.

TG measurements

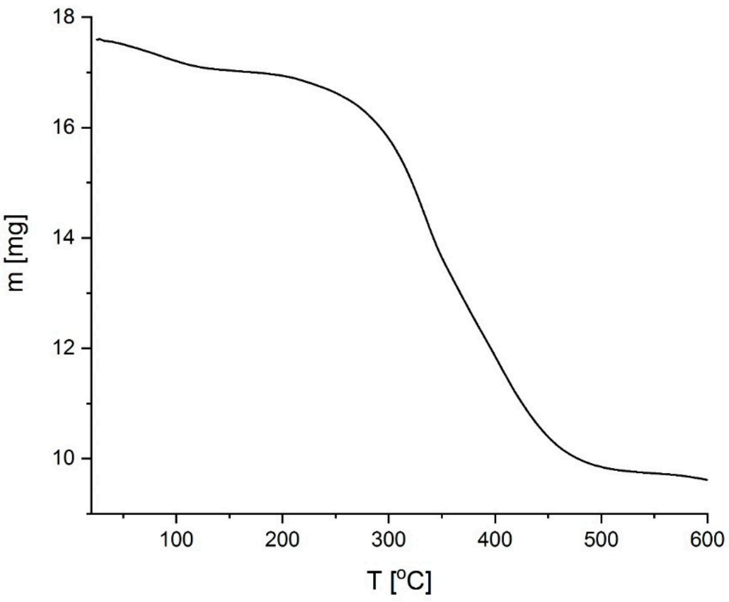

Figure S1. TG curve for complex **1**. 10 °C/min, Ar atmosphere.

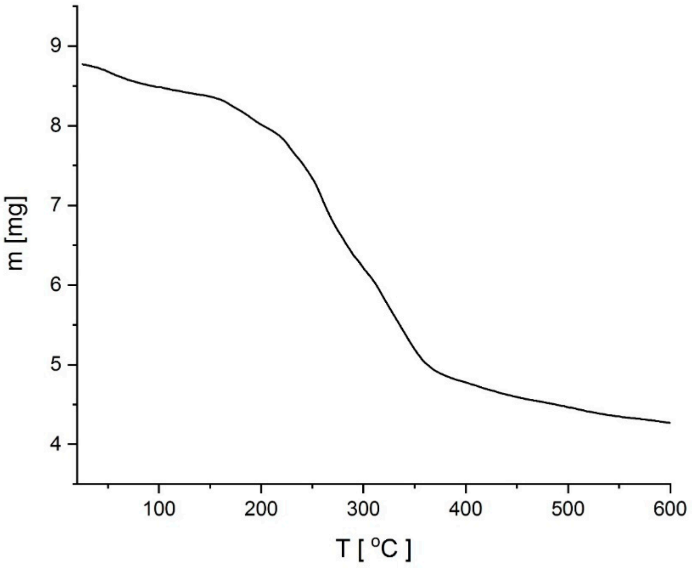

Figure S2. TG curve for complex **2**. 10 °C/min, Ar atmosphere.

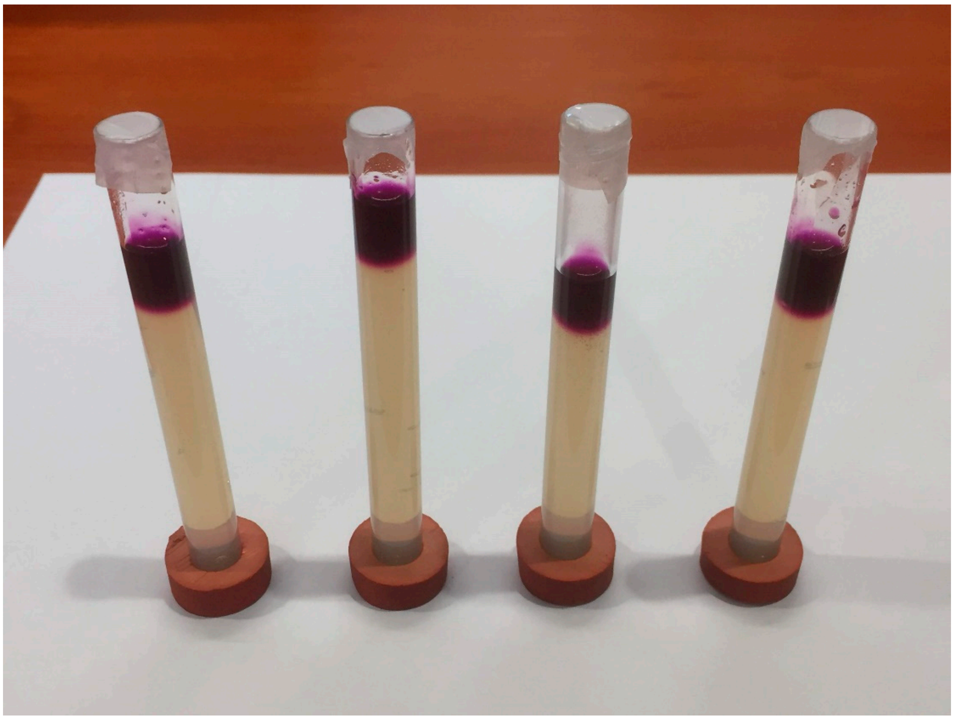

Figure S3. Photo of single crystal growth assembly.

Table S1. The structural data

		1	2	3
**Empirical formula**		$C_{26}H_{16}ClN_{10}WZn$	$C_{26}H_{18}CdN_{11}O_4W$	$C_{26}H_{20}CdN_{10}O_3W$
**Formula weight**		753.16	844.76	816.77
**Temperature [K]**		100(2)	273(2)	250(2)
**Wavelength [Å]**		0.071073	0.71073	0.71073
**Crystal system**		Triclinic	Triclinic	Monoclinic
**Space group**		P -1	P -1	P 2_1/c
**Unit cell dimensions**	**a [Å]**	8.16770(10)	7.1595(3)	12.4802(2)
	**b [Å]**	10.20350(10)	11.7648(7)	15.8430(2)
	**c [Å]**	16.3674(3)	17.4816(9)	14.8743(2)
	**α [°]**	96.4850(10)	73.389(5)	90
	**β [°]**	95.9790(10)	88.087(4)	108.215(2)
	**γ [°]**	107.8160(10)	74.562(4)	90
**Volume [$Å^3$]**		1276.37(3)	1358.66(13)	2793.63(7)
**Z**		2	2	4
**Density (calculated) [Mg/m^3]**		1.960	2.065	1.942
**Absorption coefficient [mm^{-1}]**		5.585	5.069	4.923
**F(000)**		726	810	1558
**Crystal size [mm^3]**		0.200 × 0.150 × 0.050	0.200 × 0.150 × 0.100	0.150 × 0.100 × 0.100
**Theta range for data collection [°]**		2.121 to 32.309	2.955 to 28.672	2.858 to 30.140
**Index ranges**		$-11 \le h \le 12$<br>$-14 \le k \le 15$<br>$-24 \le l \le 24$	$-9 \le h \le 8$<br>$-14 \le k \le 14$<br>$-23 \le l \le 23$	$-17 \le h \le 17$<br>$-22 \le k \le 21$<br>$-20 \le l \le 19$
**Reflections collected**		66658	9571	36434
**Independent reflections**		8154 [R_{int} = 0.0564]	6052 [R_{int} = 0.0489]	7723 [R_{int} = 0.0401]
**Completeness to theta [%]**		99.8	99.7	99.9
**Refinement method**		Full-matrix least-squares on F^2	Full-matrix least-squares on F^2	Full-matrix least-squares on F^2
**Data / restraints / parameters**		8154 / 0 / 352	6052 / 0 / 396	7723 / 3 / 378
**Goodness-of-fit on F2**		1.076	1.050	1.075
**Final R indices [I>2σ(I)]**		R_1 = 0.0264<br>wR_2 = 0.0557	R_1 = 0.0499<br>wR_2 = 0.1152	R_1 = 0.0263<br>wR_2 = 0.0511
**R indices (all data)**		R_1 = 0.0313<br>wR_2 = 0.0569	R_1 = 0.0663<br>wR_2 = 0.1265	R_1 = 0.0398<br>wR_2 = 0.0565
**Largest diff. peak and hole [e/$Å^3$]**		1.336 and -1.623	1.834 and -2.434	1.268 and -0.645

EDS measurements

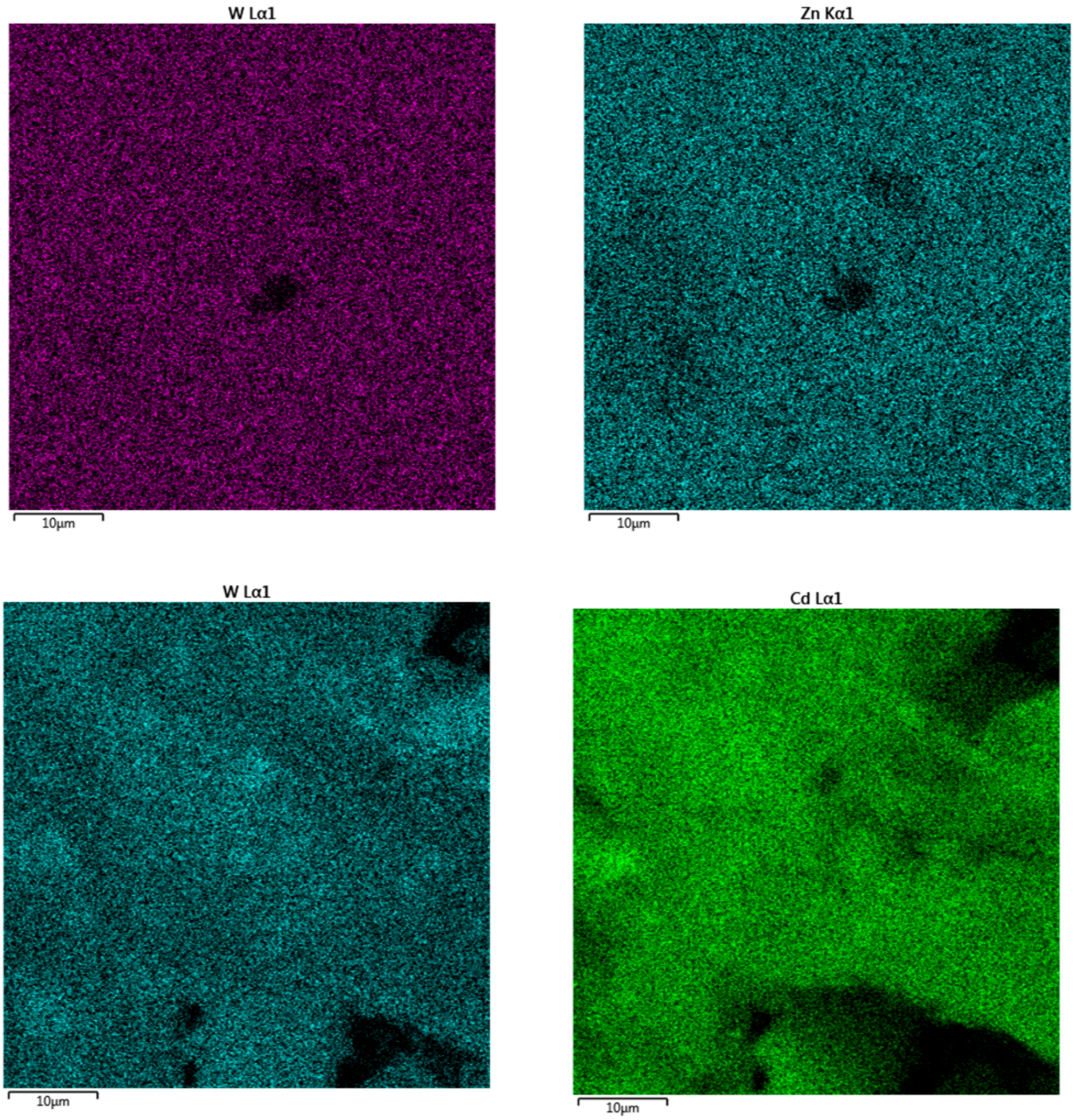

Figure S4. Scanning electron microscope photographs of **5** (up) and **6** (down) with W and Zn or Cd distribution.

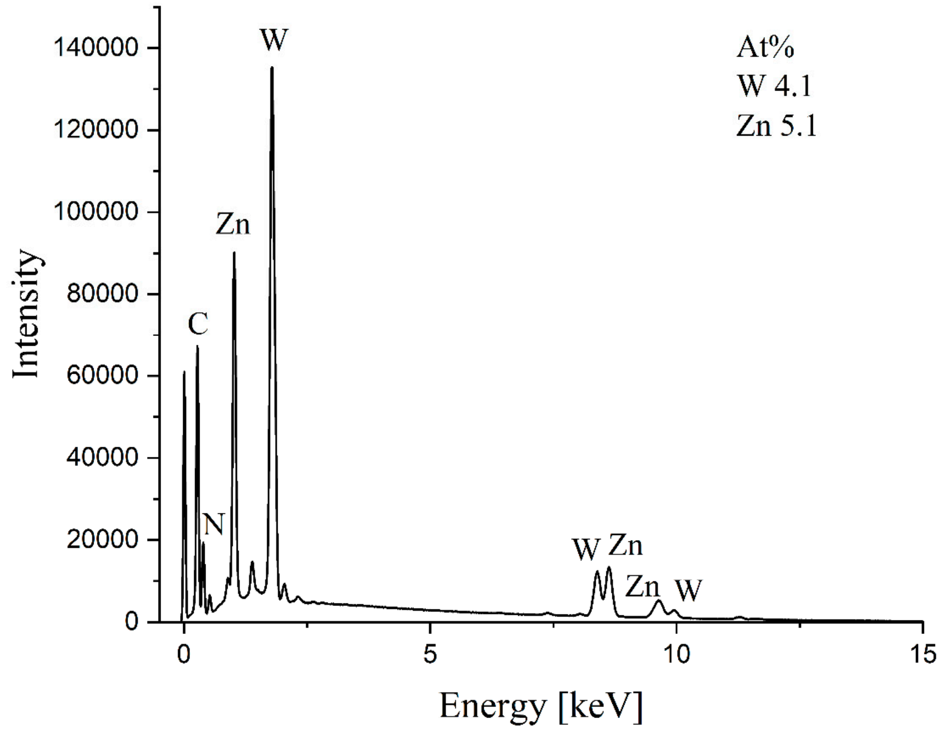

Figure S5. EDS spectrum of **5**.

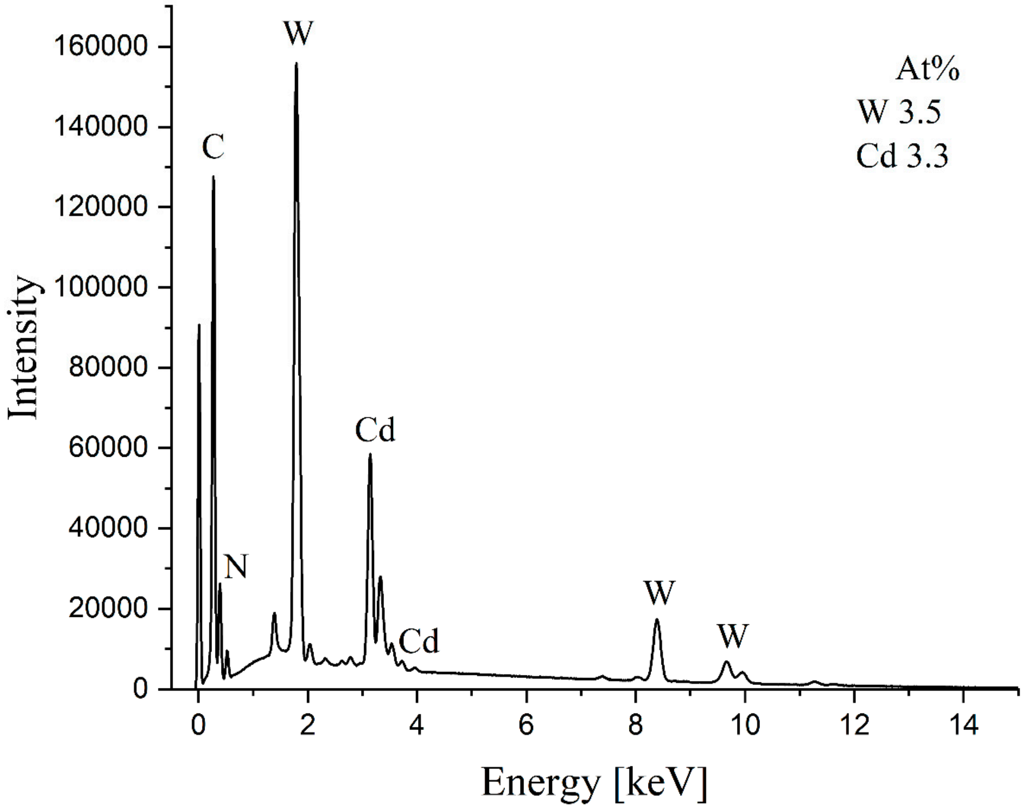

Figure S6. EDS spectrum of **6**.
